# Static electricity passively attracts ticks onto hosts

**DOI:** 10.1016/j.cub.2023.06.021

**Published:** 2023-06-30

**Authors:** Sam J. England, Katie Lihou, Daniel Robert

**Affiliations:** 1 School of Biological Sciences, Faculty of Life Sciences, University of Bristol, 24 Tyndall Avenue, BS8 1TQ Bristol, UK; 2 Museum für Naturkunde – Leibniz Institute for Evolution and Biodiversity Science, Invalidenstraße 43, 10115 Berlin, Germany

## Abstract

Most terrestrial animals naturally accumulate electrostatic charges, meaning that they will generate electric forces that interact with other charges in their environment, including those on or within other organisms. However, how this naturally occurring static electricity influences the ecology and life history of organisms remains largely unknown.^1^ Mammals, birds, and reptiles are known to carry appreciable net electrostatic charges, equivalent to surface potentials on the order of hundreds to tens of thousands of volts.^1–7^ Therefore, we hypothesize that their parasites, such as ticks, are passively attracted onto their surfaces by electrostatic forces acting across air gaps. This biophysical mechanism is proposed by us to assist these ectoparasites in making contact with their hosts, increasing their effective “reach” because they are otherwise incapable of jumping. Herein, experimental and theoretical evidence show that the tick *Ixodes ricinus* (Figure 1A) can close the gap to their hosts using ecologically relevant electric fields. We also find that this electrostatic interaction is not significantly influenced by the polarity of the electric field, revealing that the mechanism of attraction relies upon induction of an electrical polarization within the tick, as opposed to a static charge on its surface. These findings open a new dimension to our understanding of how ticks, and possibly many other terrestrial organisms, find and attach to their hosts or vectors. Furthermore, this discovery may inspire novel solutions for mitigating the notable and often devastating economic, social, and public health impacts of ticks on humans and livestock.^8–15^

## Results

### Ticks are attracted by electrostatically charged animal fur

**Figure 1:**
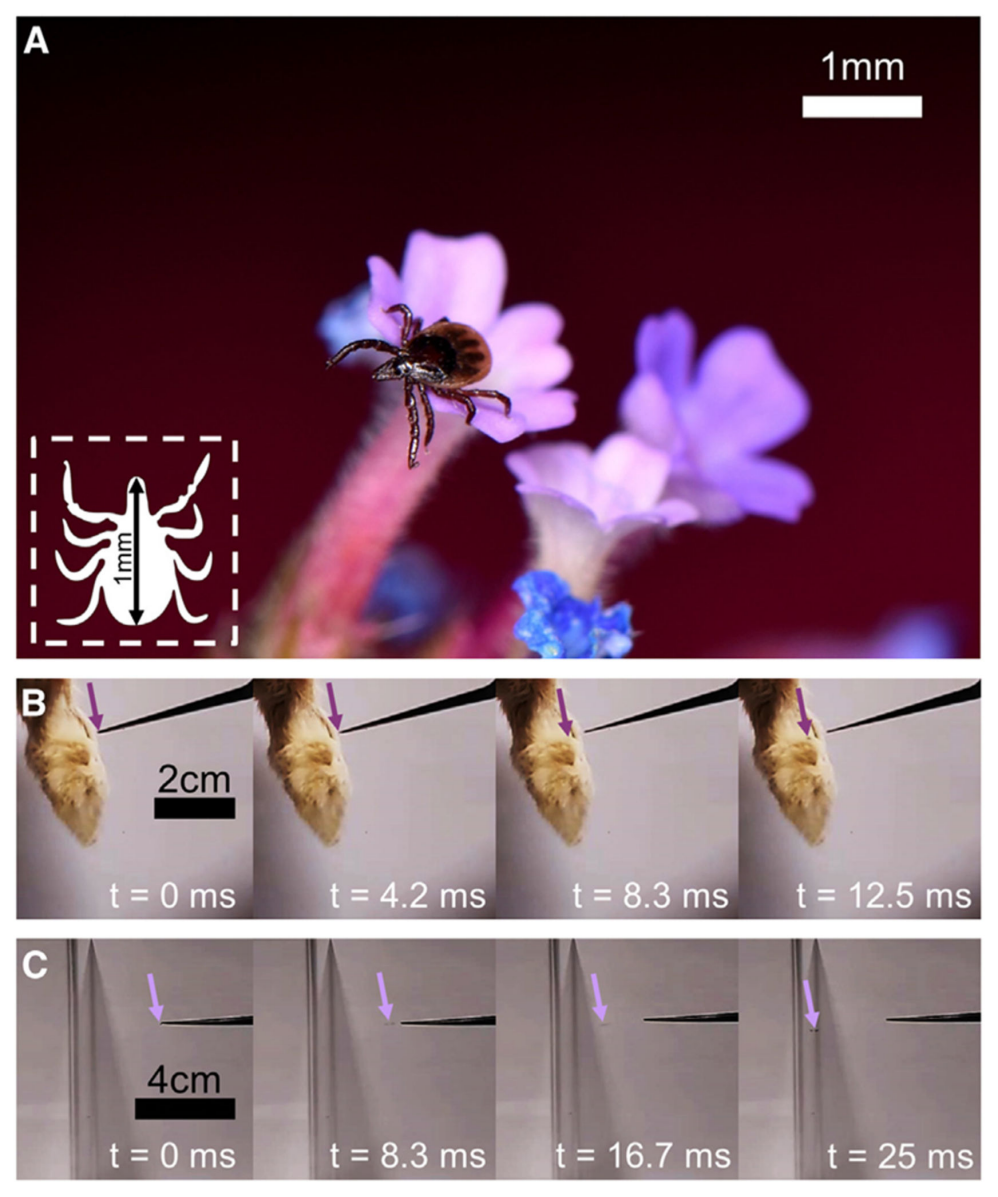
*Ixodes ricinus* and its ability to be attracted by static electric fields (A) Macrophotograph of an *I. ricinus* nymph. (B) Frames from a 240-fps video recording of a tick passively attracted by the static charge of a rabbit foot across an air gap of several millimeters. Purple arrows indicate tick location. See Video S2. (C) 240-fps video frames of a tick passively attracted by the static charge of an acrylic sheet triboelectrically charged with rabbit fur across an air gap of several centimeters. Pink arrows indicate tick location. See Video S3. See also Video S1 for electrostatic attraction of a freely walking tick.

First, to initially test the capacity of charged animals to attract ticks with electrostatic forces, live *I. ricinus* nymphs (typical length ≈ 1 mm, typical mass ≈ 0.1 mg^16,17^) were held with stainless steel entomological forceps and brought into the proximity of electrically charged rabbit feet and acrylic surfaces triboelectrically charged by rabbit fur. Rabbit feet were triboelectrically charged to an average net charge magnitude of 752 ± 679 pC (mean ± SD, N = 20). Ticks were readily attracted across air gaps of up to several millimeters or centimeters onto these statically charged surfaces (Figures 1B and 1C; Videos S2 and S3). This establishes that electrostatic attraction of ticks onto hosts can take place over large air gaps of many body lengths of a tick.

### Strong electric fields exist between tick hosts and vegetation

**Figure 2:**
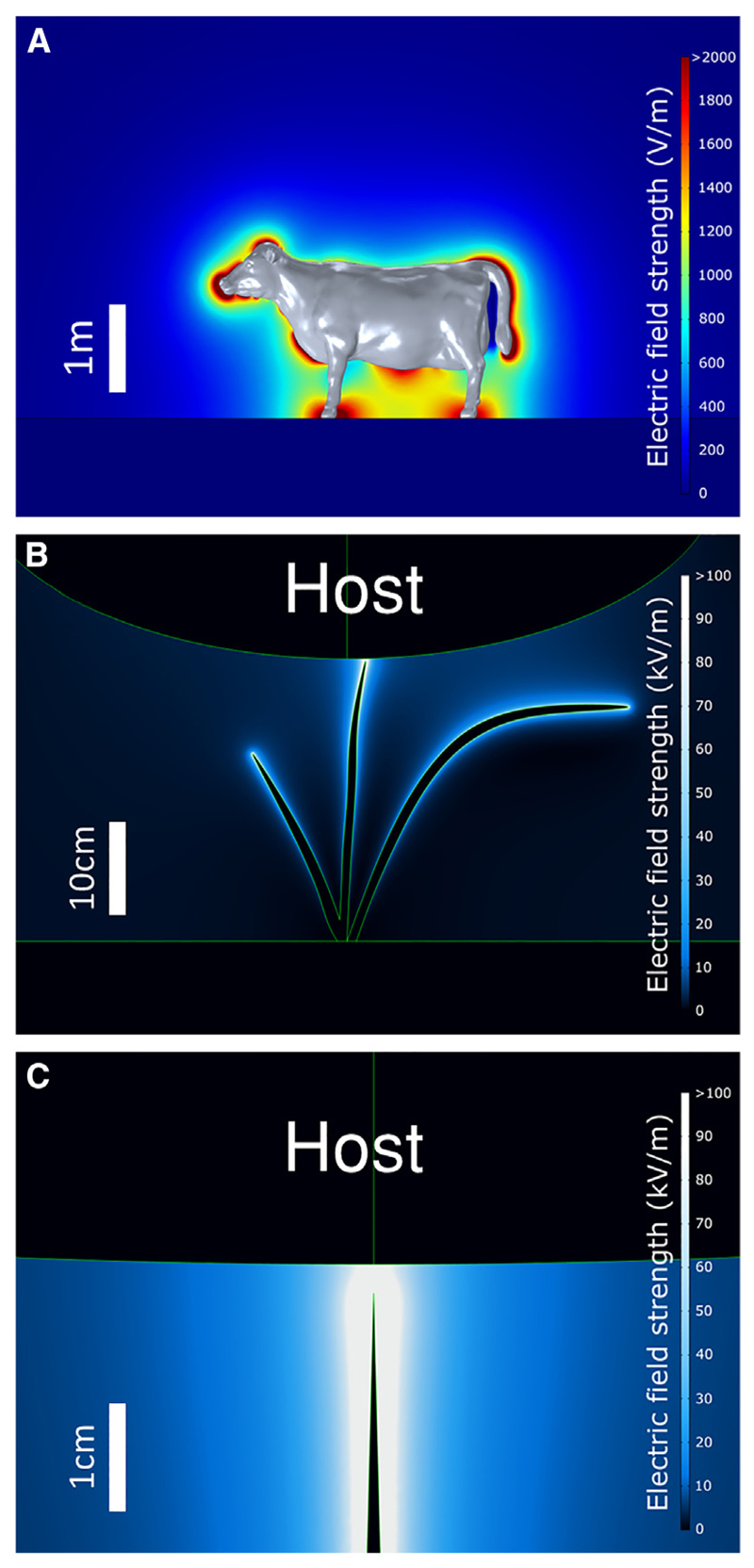
Mapping electrostatic interactions between tick hosts and vegetation Three-dimensional finite element models of electric fields around a host and between host and vegetation. Vegetation is electrically grounded, hosts have a uniform surface potential of +750 V. See Table S1 for model input parameters. (A) Electric field strength surrounding a charged cow. (B) Electric field strength between a charged host and grounded tuft of grass. (C) Electric field strength between a charged host and grounded blade of grass, separated by 2.5 mm.

To evaluate the electrostatic footprint of the surface charges of typical tick hosts in the natural environment, we produced several computational finite element models that estimate the structure and strength of the electric interactions between charged hosts and the surrounding vegetation upon which ticks quest. These models show that the environment of a tick is electrically vibrant. Taking a cow as an example, assuming a uniform +750 V potential across its surface (a conservative estimate for the surface voltage attainable by a vertebrate^1–7^), this modeling highlights significant hotspots of electric field, with strong regions around the nose, tail, and limbs (Figure 2A). Notably, these body parts are the most likely to be encountered by ticks questing in vegetation. Next, the examination of the electric field around grounded vegetation in proximity to a charged animal reveals considerable electric field strengths in the first few millimeters away from the vegetation (Figures 2B and 2C). Looking even closer, the electric field strength 0.5 mm away from the vegetation (approximately the midpoint of the space that would be occupied by a *I. ricinus* nymph) can reach in excess of 300 kV/m (Figure 2C).

### Live ticks are passively attracted by the electric fields of their hosts

**Figure 3:**
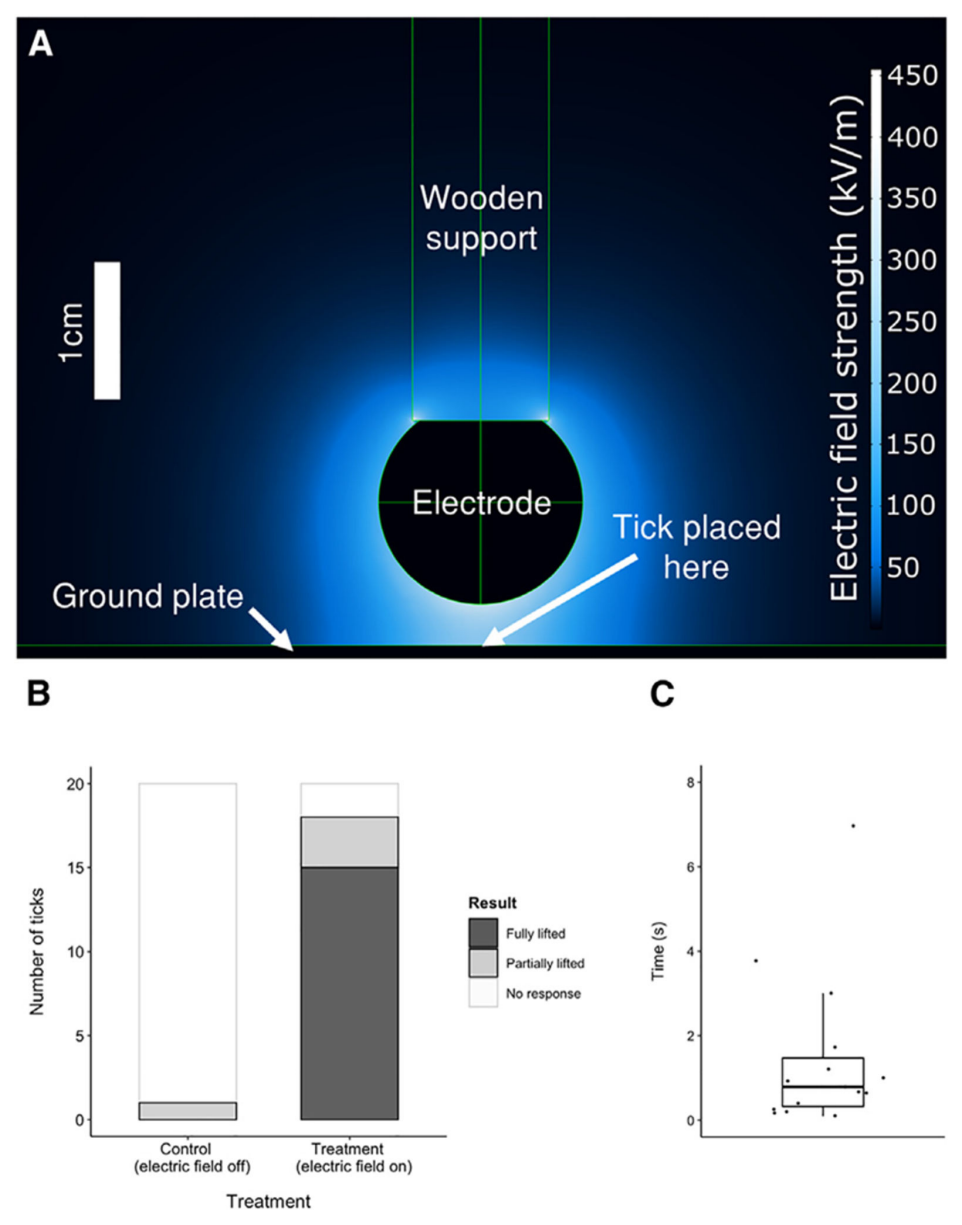
Number and timing of live ticks attracted electrostatically across a 3 mm air gap against gravity (A) Model of experimental apparatus and electric fields (strength as color gradient). Field strength data are generated computationally using the finite element method. See Table S1 for model parameters. (B) Number of live ticks fully or partially lifted in treatment and control conditions. In the control group, no voltage (0 V) was applied to the electrode and in the treatment group, +750 V was applied. (C) The time taken for each tick to fully lift off from the ground plate following electric field onset (median time = 0.79 s; IQR = 2.05 s). Midline of boxplot is the median, edges of box are the first and third quartiles, and whiskers are the minimum and maximum, defined by the first and third quartiles ± 1.5 × interquartile range.

Based upon these modeled electric field strengths, attraction between ticks and their hosts was investigated in controlled empirical experiments. Live *I. ricinus* nymphs were placed on an electrically grounded aluminum plate, directly beneath a steel spherical electrode (diameter = 1.5 mm, 3 mm from ground plate) (Figure 3A). In treatment trials (n = 20), a voltage of +750 V was supplied to the electrode for 20 s, mimicking the electric field of a host situated a few millimeters away from the grounded plant surface. In control trials (n = 20), no voltage was supplied, and the electric potential between sphere and plate was set to zero by shunting. It was then observed whether each tick was fully lifted across the air gap, leaving the ground plate, traveling through the air against gravity, and making contact with the electrode. Some ticks were only partially lifted across the air gap, maintaining contact with the ground plate and thus did not reach the electrode. This partial lifting was also noted because these ticks were clearly also subjected to significant forces by the electric field but were resisting its pull by holding on to the ground plate.

Ticks were only fully lifted across the air gap when the electrode was switched on, and never when it was turned off (Figure 3B). In the presence of the electric field (treatment group), 75% (CI ± 3.8%; n = 15) of the ticks were fully lifted to the electrode, and 15% (CI ± 1.7%; n = 3) of ticks were partially lifted. When no electric field was applied (control group), 0% (CI ± 0.0%; n = 0) of ticks were fully lifted to the electrode, and 5% (CI ± 0.98%; n = 1) of ticks partially lifted towards the electrode. The proportion of ticks that fully lifted to the electrode was significantly higher in the treatment group, than in the control group (χ^2^ = 29.6; N = 40, P < 0.0001; post-hoc: *residuals* = 4.6, *P* < 0.0001). The singular tick that partially lifted towards the electrode in a control trial, as judged by an observer in a blind scoring protocol, met the criteria by tilting suddenly to one side, doing so under its own volition in the process of locomotion. This movement was visually indistinguishable from the ticks that were partially lifted by the presence of an electric field and so was included for consistency. Ticks that were fully lifted across the air gap left the ground plate within seconds of voltage onset (median = 0.79 s; IQR = 2.05 s; Figure 3C). This experiment demonstrates that electric fields arising from a surface voltage commensurate with those reported for vertebrates were sufficient to pull tick nymphs across air gaps of several body lengths and against gravitational attraction. Thus, electrostatic forces have the capacity to greatly increase the spatial range and efficiency of host finding.

It is worth noting that in this experimental paradigm, the electrostatic force lifts the ticks in direct opposition to the force of Earth’s gravity. In nature, however, the host may be situated in any direction, implying other directions of travel would require lesser electric field strengths than quantified in this experiment.

Further, whilst both the substrate and electric field source were conductive in this experiment due to the necessity to finely control their voltages, the same powerful electrostatic attraction was also seen for ticks on insulating substrates exposed to triboelectrically charged insulators (Video S1). Therefore, our findings likely hold for ticks inhabiting a wide variety of habitats and vegetation types, with different electrical properties.

### Determining the threshold of electrostatic tick attraction

To establish the minimum electric field strength required to elicit electrostatic attraction of ticks, individual *I. ricinus* nymphs were placed on the ground plate directly underneath the spherical electrode positioned at six different distances from the ground plate, ranging from 1.5 to 4 mm in 0.5 mm increments. Twelve ticks were tested for each distance (N = 72). The threshold voltage required for tick attraction was determined at each distance by holding the electrode at a low positive starting voltage for 20 s; if the tick was not fully lifted to the electrode during this time, the electrode was disconnected for 20 s, and then the voltage was increased in magnitude by 50 V and reconnected for a further 20 s, again observing whether the tick was fully lifted to the electrode. This process was repeated, in increments of 50 V, until the tick was electrostatically pulled across the air gap onto the electrode. This critical voltage was deemed as the threshold for that individual tick at that distance. To avoid the spurious influence of ticks choosing to hold on to the ground plate, freshly dead ticks were used in this and all subsequent experiments. Each trial was conducted within 30 min of expiration to minimize the impact of desiccation on the electric properties of the ticks.

**Figure 4:**
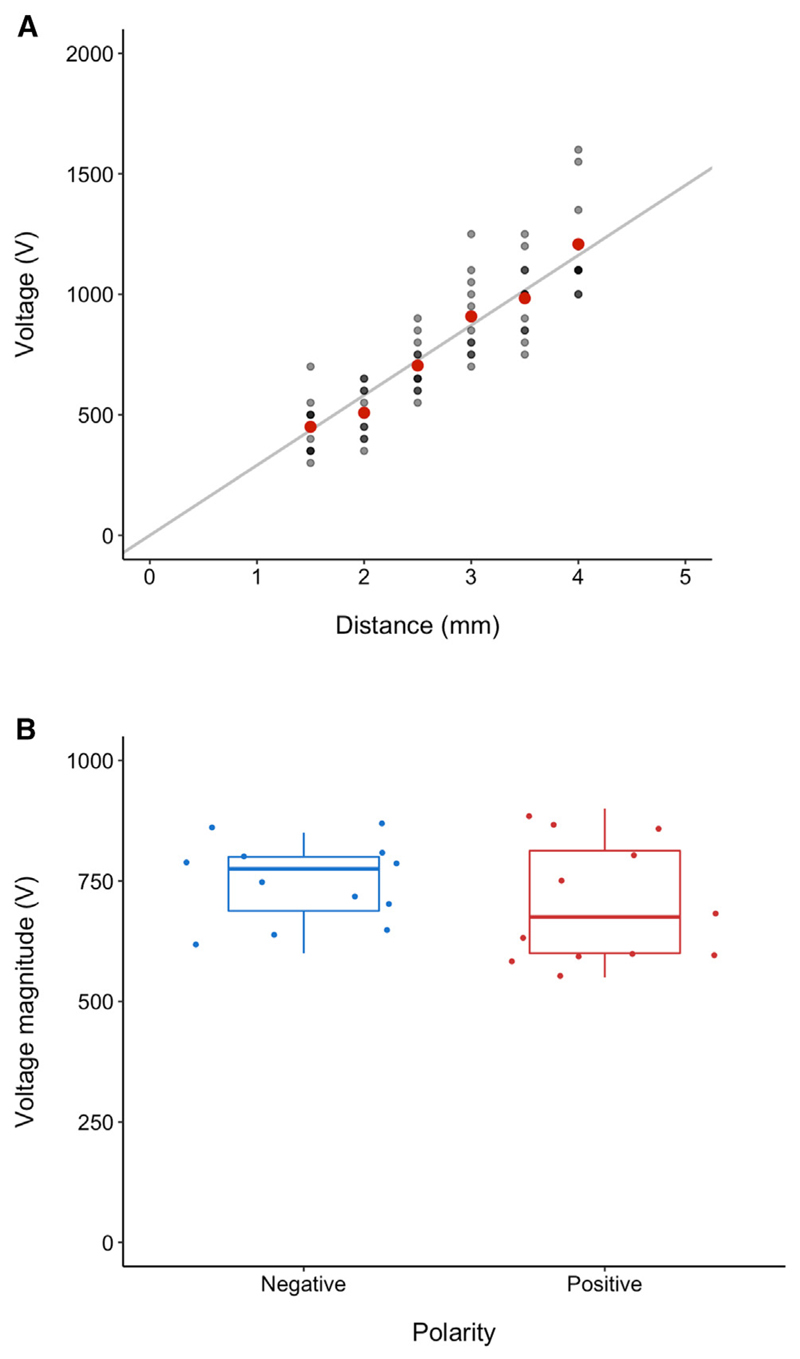
Threshold and mechanism of electrostatic tick attraction (A) The relationship between distance to the electrode (mm) and the voltage (V) required to fully lift freshly dead ticks across air gaps against gravity. Light gray points indicate one data point at value, dark gray indicates two data points, and black indicates three data points. Red points show the mean voltage for each distance measured. Straight line shows the linear regression model with forced zero intercept (R^2^ = 0.97, F_1,71_ = 2371.1, P < 0.0001). (B) Voltage magnitudes (V) required to lift dead ticks across a 2.5 mm air gap against gravity for both positive and negative voltages. n = 12 for each group. Midline of boxplots is the median, edges of boxes are the first and third quartiles, and whiskers are the minimum and maximum, defined by the first and third quartiles ± 1.5 × interquartile range.

The relationship between the distance of electric field source and the voltage required to fully lift the tick across the gap shows a strong linear trend (Figure 4A). This is expected because the electric force on a charged object is proportional to the electric field strength, i.e., the voltage per distance. Therefore, because the gravitational force on the ticks should be essentially identical in all trials, and their apparent charges are likely similar, the electric force (and thus the electric field strength) required to lift the ticks against gravity should be approximately the same across all distances. The distance between the tick and the electrode had a significant positive effect on the voltage required to attract ticks to the electrode (general linear model with forced zero intercept, *R*^2^ = 0.97, *F*_1,71_ = 2371.1, P < 0.0001; Figure 4A). The gradient of this line is 290 kV/m, which is a slight overestimation of the average electric field strength required because the experimental apparatus did not generate perfectly uniform electric fields (Figure 3A). Computational modeling of the experimental apparatus with the average experimental voltage required for each distance applied to the electrode concurs with this result, suggesting that the average electric field strength required for tick attraction across the range of distances tested was 258 kV/m. This threshold allows for extrapolation to smaller and larger voltages and distances. For example, if a tick is to be attracted across a much smaller gap of 0.1 mm, then a much lower voltage of around 26–29 V is required. Even being lifted across small sub-millimeter gaps such as this will provide an improvement to ticks’ host-finding efficiency while necessitating only very small and easily accumulated voltages on the host. It is also worth considering here that humans are reportedly capable of accumulating surface voltages as high as 30,000 V.^2,3^ Utilizing our empirical voltage-distance relationship, a 30 kV surface voltage could theoretically attract *I. ricinus* nymphs across a gap of several centimeters, highlighting the potentially considerable influence of static electricity on parasite ecology. Crucially, the computational models of the electric field strength surrounding vegetation approached by a terrestrial vertebrate (Figure 2) show that our experimentally determined threshold electric field strength is reached in realistic conditions. Altogether, the evidence supports the hypothesis that ticks in nature will be exposed to electric fields of sufficient strength to lift them onto their hosts. Importantly, the electric field strengths around vegetation exposed to the vertical atmospheric potential gradient, as is found in open ground,^7,18–23^ are well below the threshold required for tick attraction. Therefore, ticks will only be electrostatically pulled from vegetation by the presence of a potential host, and not by ambient atmospheric electric fields. Ticks are therefore not expected to utilize the atmospheric potential gradient to disperse, unlike spiders.^22,24,25^

### Elucidating the mechanism: Positive vs. negative voltages

To unveil the mechanism of electrostatic tick attraction, we determined whether attraction was dominated by static surface charge or by an induction of electric polarization within the tick. To do this, we compared attraction thresholds toward electrodes of different polarities (n = 12 with positive voltage, n = 12 with negative voltage). In effect, if the surface charge of the tick is non-negligible and static in this interaction, the ticks would exhibit a greater degree of attraction to the electrode polarity opposite to their own net charge. Conversely, if ticks are equally attracted to both positive and negative voltages, we can infer that the attraction mechanism must be primarily relying upon the induction of a polarization or charge separation (through dielectric polarization and/or image charging) within the tick.

There was no significant difference between the median voltage magnitude at which ticks were attracted to the electrode between negative (775 V [CI 750–875]) and positive (675 V [525–750]) voltages (*W* = 88.5, P = 0.4; Figure 4B). This result strongly indicates that the mechanism of attraction is dominated by an induction of polarization or charge separation within the tick, as opposed to relying upon a quasi-fixed, or permanent, electrostatic charge on the surface of the tick. The ultimate consequence of this is that ticks are expected to be equally attracted to both positively and negatively charged animals in nature, further increasing their host-seeking abilities.

## Discussion

Altogether, we have shown here that static electric fields produced by tick hosts are sufficient to passively attract ticks across air gaps of several millimeters and more. Remarkably, the electric force generated is strong enough to overcome the action of gravity, readily enabling lateral or vertical lifting motions. In nature, we anticipate that this physical effect increases the effective reach of ticks because they have no mechanism otherwise by which to make contact with hosts beyond the reach of their bodies, such as jumping. Our results show that electrostatically charged hosts passing within a few millimeters of a tick, but without making direct contact, can generate electric conditions that enhance the capacity of ticks to successfully bridge the gap and establish contact. It is also expected that electrostatic attraction helps secure initial attachment of ticks to the host’s surface, allowing more contact time for the ticks’ tarsal claws to grip onto the host. While only ticks in their nymphal stage were used in the experiments of this study, preliminary investigations on larval and adult ticks (which are several times smaller and larger than nymphs, respectively^16,26–28^) indicated that they too can be passively attracted by electric fields of the same magnitude. Therefore, electrostatic attraction onto hosts is likely ontogenetically omnipresent in ticks.

Based on the mechanisms and thresholds elucidated by this study, it is highly likely that passive electrostatic attraction is not limited to ticks and may be widespread among other terrestrial ectoparasitic arthropods, such as mites, fleas, or lice. Indeed, it has previously been hypothesized that the honeybee parasitic mite *Varroa destructor* is passively attracted by the static charge accumulated on honeybees.^29,30^ The findings of our study provide empirical evidence in support of that hypothesis.

Ticks and other terrestrial ectoparasites also engage in phoresy, wherein they “hitchhike” on other species, typically insects, purely for the purpose of dispersal.^31–36^ As insects are also well known to carry electrostatic charge,^1^ this ecological interaction may similarly be enabled or facilitated by passive electrostatic attraction.

Furthermore, our finding that naturally occurring electric fields are ecologically relevant to ticks suggests that the detection of these electric fields could provide an advantageous sensory cue during the host-finding process. This would be an example of aerial electroreception, a newly discovered sensory modality that has already been demonstrated in other arachnids and arthropods.^1,7,22,25,37–40^ Ticks use many different sensory modalities to detect the presence of their hosts.^41–44^ Questing in *I. ricinus* can be triggered by multiple different stimuli, including vibrations, radiative heat, host smell, and a sudden fall in light intensity.^41^ The Haller’s organ, located on the forelegs, is the primary sensory structure utilized for host seeking in ticks and includes humidity receptors, chemoreceptors, carbon dioxide and temperature sensilla, along with tactile filiform bristles on the foreleg tibia.^41^ It is possible that some of these sensilla, particularly the filiform bristles, could be co-opted for electroreception. Ticks also possess mechanoreceptive trichobothria, a sensory structure that in other arachnids has previously been shown to be sensitive to electric fields.^22,45^ Altogether, it is conceivable that changes in local electric field, caused by the electrostatic charge of approaching hosts, may constitute an additional cue used by ticks to initiate questing or other host-seeking behaviors. The possibility of electroreception as a sensory ability exhibited by ticks warrants immediate investigation and would provide a novel ecological context for electroreception in air: parasitism.

Lastly, it is important to consider the potential applications of this research. Because electrostatic charge has been unveiled as a mediator for tick attraction, strategies and technologies can now be developed to disrupt this electrical interaction. For example, the treatment of livestock, pets, or human clothing with anti-static coatings may well reduce the rates of tick infestation in these contexts. Indeed, many synthetic fiberes readily accumulate electrostatic charge at much higher magnitudes than natural materials.^1,46,47^ Conceivably, outdoor clothing made from fibers with minimal or nil capacity for electrostatic charging, or treated with anti-static coatings, could reduce encounters with ticks.

Overall, this study has unveiled a previously unknown mechanism by which ticks and possibly many other terrestrial ectoparasites are attracted onto their hosts, opening up new research avenues aimed at mitigating the effect of ticks and tick-borne diseases in nature and society.

## Star⋆Methods

Detailed methods are provided in the online version of this paper and include the following:

- KEY RESOURCES TABLE
- RESOURCE AVAILABILITY
  ○ Lead contact
  ○ Materials availability
  ○ Data and code availability
- EXPERIMENTAL MODEL AND SUBJECT DETAILS
- METHOD DETAILS
  ○ Attraction of ticks to charged animal fur and surfaces
  ○ Computational modeling of electric fields
  ○ Electrostatic attraction of live ticks with voltages
  ○ Threshold electric field strengths: The distance-voltage relationship
  ○ Elucidating the mechanism: Positive vs. negative voltages
  ○ Quantification and statistical analysis

## Star⋆Methods

### Key Resources Table

**Table:** 

REAGENT or RESOURCE	SOURCE	IDENTIFIER
Deposited data		
Experimental data	This paper	Mendeley Data: https://doi.org/10.17632/kthkkwkhmp.1
Experimental models: Organisms/strains		
*Ixodes ricinus*	Wild-caught in Leigh Woods, Bristol, United Kingdom (approx. 51°27′47″N 2°38′21″W)	Wild type
Software and algorithms		
R v. 3.6.1	R Core Team	https://www.r-project.org/
COMSOL Multiphysics® v. 5.4	COMSOL AB, Stockholm, Sweden	https://www.comsol.com/comsol-multiphysics
BORIS	Friard and Gamba^48^	http://www.boris.unito.it/
Other		
JCI 147 Faraday pail	Chilworth Technology Ltd., Southampton, United Kingdom	N/A
JCI 140C static monitor	Chilworth Technology Ltd., Southampton, United Kingdom	N/A
PS350/5000V-25W high voltage power supply	Stanford Research Systems, Inc., Sunnyvale, CA, United States	N/A
Nikon D3400 DSLR camera	Nikon Corporation, Tokyo, Japan	https://www.nikonusa.com/en/nikon-products/product/dslr-cameras/d3400.html
F-S DX Micro NIKKOR 85mm f/3.5G ED VR lens	Nikon Corporation, Tokyo, Japan	https://www.nikon.co.uk/en_GB/product/lenses/dslr/af-s-dx-micro-nikkor-85mm-f3.5g-ed-vr
Google Pixel 4A	Google Inc., Mountain View, California	N/A

### Resource Availability

#### Lead contact

Further information and requests should be directed to and will be fulfilled by the lead contact, Sam J. England (sam.england@mfn.berlin)

#### Materials availability

This study did not generate new unique reagents.

#### Data and code availability

- All data have been deposited in Mendeley Data and are publicly available as of the date of publication. The DOI is listed in the key resources table.
- This paper does not report original code.
- Any additional information required to reanalyze the data reported in this paper is available from the lead contact upon request.

### Experimental Model and Subject Details

Wild *Ixodes ricinus* ticks were collected from Leigh Woods, Bristol, United Kingdom (approx. 51° 27′47″ N 2°38′21″W) between April and October 2021 by dragging white cotton sheets through low-lying vegetation. These ticks were then maintained at 7°C in sample tubes containing water-soaked filter paper to maintain humidity, until experimentation was conducted. Prior to inclusion in an experiment, ticks were allowed to acclimatise to laboratory conditions for a minimum of 1 h. Ticks were then checked for vitality before experimentation by seeing if they visibly walked on a substrate. *I. ricinus* has three life stages in which it feeds on hosts: larvae, nymph, and adult, however due to the numbers of each lifecycle stage collected, all ticks used in the formal experiments were nymphs.

### Method Details

#### Attraction of ticks to charged animal fur and surfaces

To initially test whether live ticks would be attracted to electrostatically charged animal fur and surfaces charged by animal fur, live *I. ricinus* nymphs (typical length ≈ 1 mm, typical mass ≈ 0.1 mg^16,17^) were held in stainless steel entomological forceps and brought into the proximity of triboelectrically charged rabbit feet or acrylic. Dried rabbit feet were charged by friction with acrylic. Subsequent tests showed that rabbit feet charged by the same protocol possessed an average net charge magnitude of 752 ± 679 pC (mean ± SD, N = 20), as measured by dropping individual rabbit feet into a Faraday pail and static monitor system (JCI 147 & JCI 140C, Chilworth Technology Ltd., Southampton, United Kingdom). The acrylic was charged by friction with rabbit fur, and therefore should carry a charge equal in magnitude but opposite in polarity to the rabbit fur. Upon being brought to horizontal distances of a few millimeters to centimeters from the charged fur or acrylic, the grip of the forceps was loosened, and any subsequent attraction of the ticks was filmed at a frame rate of 240 fps (Google Pixel 4A, Google Inc., Mountain View, California). Because electrostatic attraction was also seen in ticks walking freely on an insulating surface (Video S1), any attraction seen here is evidently not symptomatic of the ticks being held and released from the conductive forceps.

#### Computational modeling of electric fields

Computational modeling of electric fields was performed using three-dimensional finite element analysis within COMSOL Multiphysics® v. 5.4 (COMSOL AB, Stockholm, Sweden). Each model utilized the Electric Currents interface within the AC/DC module. For this interface, the electrical conductivity, *σ*, and relative permittivity, ε_*r*_, for each material must be defined (see Table S1). Model outputs are presented as 2D slices through the 3D datasets. Details specific to each model are given below.

#### Model of electric field around a cow

The geometry of this model was contained within a 12 × 12 × 12 m cube. The bottom 2 m of this cube were filled by a 12 × 12 × 2 m cuboid representing the soil. Atop this soil layer was located a detailed 3D representation of a cow. The height of the cow was 177cm. The bottom surface of the cube, 2 m below the surface of the soil, was defined as the electrical ground, and a uniform potential of +750 V was applied to the surface of the cow, as this is a typical surface voltage found on a terrestrial animal.^2–4,6,7^ Whilst we chose a cow as a large charismatic tick host in this example here, the voltage of an object will be proportional to its charge density, and therefore the voltages of tick hosts are expected to be largely consistent across differently sized animals, if the electrical properties of their fur, scales, skin, or feathers are similar. Therefore, this model, the models subsequently described, and the voltages used in the laboratory experiments, apply to all typically charged vertebrate tick hosts. Furthermore, on the scale of a tick, both a very small host such as a mouse, and a very large host such as a cow, would both appear electrically as essentially infinitely large objects. As such, the local topography of the animal’s surface, for example the presence of hairs, is likely a much more significant influence on the electric field strength and structure than the gross geometry of the animal as a whole.

#### Models of electric field between foliage and host

The geometry of this model comprised a 1 × 1 × 1 m cube. In the lower most region of this cube lay a 1 × 1 × 0.1 m cuboid designated as the soil. On top of the soil was placed a 1 mm thick representation of a tuft of grass, approximately 40 cm tall. Then, 2.25 mm away from the upper tip of one of the grass blades was positioned an ellipsoid with semi-major axes of 0.4 × 0.4 × 0.2 m, representing the presence of an animal, given the electrical properties for cow tissue. For the magnified version of this model, a simplified grass geometry was used consisting of a singular 30 cm tall blade exactly 2.5 mm from the ellipsoid. In each case, the bottom surface of the cube, underneath the soil layer, was defined as ground, and a uniform potential of +750 V was applied to the surface of the ellipsoid representing the animal. A data probe point was specifically extracted at a distance 0.5 mm from the plant surface in the magnified model, so as to obtain the electric field strength at approximately the mid-point of the region that would be occupied by an *I. ricinus* nymph (typically about 1 mm in diameter).

#### Model of experimental setup

The geometry of this model was confined to a 0.3 × 0.3 × 0.3 m cube. The remaining geometry is based directly on precise measurements of the experimental setup. At the base of the cube lay a 200 mm × 130 mm × 1 mm aluminum plate. 3mm above this plate was positioned a spherical aluminum electrode with a diameter of 1.5 cm, this sphere intersected with a wooden cylinder (overlap 1.5 mm), with a diameter of 1 cm and a length of 15 cm. In the real-life experimental setup, this structure was supported by a micromanipulator held by a clamp-stand, however these were excluded from the model, as the distance of these objects from the tick and electrode was such that their effect on the electric field surrounding the tick would be negligible. The bottom surface of the cube, along with the plate, were defined as electrical ground (0 V), whilst the spherical electrode was assigned a potential of +750 V. Variations of this model were also created with the electrode positioned at varying heights and voltages, within the ranges seen in the experiment determining the distance-voltage relationship for tick attraction. For each height, the average threshold voltage for that height was assigned to the electrode. Data probe points were extracted at 0.5 mm from the bottom plate, to obtain the electric field strength at the mid-point of the region occupied by the tick. These electric field strengths were then averaged to calculate the average threshold electric field strength for tick attraction.

#### Electrostatic attraction of live ticks with voltages

To test the ability of the computationally predicted electric fields to passively attract ticks, live ticks were placed on a grounded conductive aluminum plate (200 mm × 130 mm × 1 mm). A conductive metal spherical electrode (diameter = 1.5 cm), controlled with a micromanipulator, was then suspended at a height of 3 mm from the plate, directly above the tick. For treatment trials, a voltage of +750 V (a reasonable estimate for the surface voltage found on a terrestrial animal^2–4,6,7^), supplied by a high voltage power source (Model PS350/5000V-25W, Stanford Research Systems, Inc., Sunnyvale, CA, United States) was then applied to the ball for 20 s. A computational model of this experimental setup indicates that the electric field strength within the first 0.5 mm above the ground plate (approximately the midpoint of the region occupied by the tick) was 222 kV/m (Figure 3A). Each trial was filmed at 50 frames per second through a F-S DX Micro NIKKOR 85mm f/3.5G ED VR lens mounted on a Nikon D3400 DSLR camera (Nikon Corporation, Tokyo, Japan), and subsequently analyzed blind to treatment or control conditions using BORIS.^48^ The temperature and humidity were recorded at the conclusion of each trial. In control trials, each of these steps were performed identically, except with the electrode connected into the ground, instead of the high voltage power source. Trials were performed in an ABBA order design for 40 trials, with no individual tick being tested more than once (n = 20 treatment ticks, n = 20 control ticks). All trials took place within an electrically shielding Faraday cage in a vibration, sound reductive, and semi-anechoic room. For analysis, it was noted whether each tick was fully lifted from the ground plate to make contact with the ball within 20 s of voltage onset, termed ‘fully lifted’. It was also recorded whether the tick appeared to be partially lifted within 20 s of voltage onset but held on to the ground plate and thus did not make contact with the ball electrode, termed ‘partially lifted’. There was no difference between median relative humidity, or variance in the relative humidity, between the control (58.5% (CI 57.0–64.5)) and treatment (58.5% (57.0–64.0)) group (*W* = 201.0, *P* = 0.989; *F*1,38 = 0.1038, *P* = 0.7491).

#### Threshold electric field strengths: The distance-voltage relationship

It was then important to quantify the influence that the distance between the tick and the electrode has on the voltage required for the tick to be fully lifted, so as to obtain a threshold electric field strength necessary for tick attraction. To reduce the spurious influence of ticks which clung onto the bottom plate, as was seen in the earlier experiment (Figure 3B), freshly dead ticks were used in this experiment. Ticks were killed by placing them into glass sample vials and then submerging these in hot water for approximately 30 s. Each tick was experimented on within 30 min of expiration to minimize the effect of drying on the electrical properties of the animal. As in the initial experiment, ticks were placed on the grounded bottom plate directly underneath the electrode, which was positioned at 6 different distances, ranging from 1.5 to 4 mm in 0.5 mm increments. Distances less than 1.5 mm could not be tested because air gaps this short could be physically bridged by the ticks in certain orientations, electrically shortcutting the system in a hazardous manner. At each distance, 12 individual ticks were tested. The threshold voltage required for tick attraction was determined at each distance by holding the electrode at a low positive starting voltage for 20 s; if the tick was not fully lifted to the electrode during this time, the electrode was disconnected for 20 s, and then the voltage was increased in magnitude by 50 V and reconnected for a further 20 s, again observing whether the tick was fully lifted to the electrode. This process was repeated, in increments of 50 V, until the tick was electrostatically pulled across the air gap onto the electrode. This critical voltage was deemed as the threshold for that individual tick at that distance, which when plotted together with each of the other ticks at all the other distances, yields a distance-voltage relationship that can be used to extrapolate to greater or lesser distances (or voltages). These threshold voltages were also inputted into a finite element analysis model of the experimental apparatus to calculate a more precise average electric field strength threshold for tick attraction, as described in the computational modeling section of the methods. The average temperature (mean ± SD) in this experiment was 21.5 ± 0.6 °C (range: 19.7–22.4 °C) and average relative humidity was 60 ± 3% (range: 50 – 64%).

#### Elucidating the mechanism: Positive vs. negative voltages

The exact mechanism by which ticks are being attracted by static electricity was investigated by testing their relative attraction to negative and positive source voltages. This is because if the attraction is based on a fixed surface charge on the tick, as is known to exist on other terrestrial arthropods,^1^ then they should only be attracted to one polarity. If, however, attraction is due to the induction of a polarization or charge separation within the tick, they should be equally attracted to both polarities. To do this, the same protocol was repeated as in the distance-voltage experiment, but with the electrode height fixed at 2.5 mm. The threshold voltage was obtained on 12 ticks for positive voltages, and a further 12 ticks for negative voltages. During this experiment, there was a significant difference in median relative humidity between the negative (35.5% (CI 35.0–36.0)) and positive (38.5% (38.0–40.5)) groups (W = 27.0, P = 0.00759), but no difference in the variance in relative humidity (F_1,22_ = 2.0494, P = 0.1663). However, to investigate any potential influence of humidity on electrostatic tick attraction, the same voltage threshold protocol described in the previous two experiments was also repeated on two groups of 12 ticks under different humidity conditions. One group were tested in ‘low humidity’ conditions ranging from 35-39% (median = 38.5% (CI 38.0–40.5)) relative humidity, and the other in ‘high humidity’ conditions ranging from 59-62% (median = 61.0% (60.5–62.0)) relative humidity. Positive voltages on an electrode at a distance of 2.5 mm were used in all trials. There was no difference in the median voltage needed to attract dead ticks to the electrode between low (675V (CI 525-750)) and high (675V (575-725)) relative humidity condition groups (Figure S1) (W = 69.5, P = 0.9069), and no difference in the voltage variance between groups (F_1,22_ = 0.4171, P = 0.5251). See Figure S1. This shows that the significant difference in humidity between the positive and negative voltage groups tested in the previous experiment, is unlikely to have affected the voltage magnitude required to fully lift the ticks.

#### Quantification and statistical analysis

All data analyses were conducted in R (v 3.6.1; R Core Team, 2021). Confidence intervals (CI) are all 95% confidence intervals, calculated as Wald’s confidence intervals for binomial data, and are bootstrapped estimates (n = 10000) of confidence intervals of the median for other non-normally distributed data. All statistical tests are two-tailed. For each experiment, *a priori* power analyses were conducted using https://www.statskingdom.com/index.html to obtain sample sizes needed for a test power of 0.8 and large effect sizes.

#### Electrostatic attraction of live ticks with voltages

A Chi Square test of independence was used to test for differences in the proportion of ticks attracted to an electrode (including ticks that ‘fully lifted’ and ‘partially lifted’) between the control and treatment group. Post-hoc comparisons of standardized residuals with Bonferroni correction, was used to compare the difference between tick that ‘fully lifted’ to the electrode and ticks that ‘partially lifted’ towards the electrode, between the control and treatment groups.

Differences in the humidity between the control and treatment group were tested using the Mann-Whitney *U* test, as the data distribution was non-normal. As the data contained ties, approximated *P*-values were calculated using the permutation method. The Levene’s test was used to compare the variance between the two groups.

#### Quantification of the relationship between distance and voltage required to attract ticks

A simple linear regression model was used to quantify the relationship between distance (independent variable) and voltage (dependent variable). As by physical definition, the intercept of this relationship must necessarily be zero, the standard model intercept confidence intervals were assessed using the *confint*.*lm()* function, to see if this could conceivably be the case. The standard linear model was compared with a linear model with a forced zero-intercept using ANOVA and *R*^2^ values. ANOVA was used to test whether the selected linear model explained significantly more of the variance than the null model (model without distance).

#### Comparison between positive and negative voltages

Differences between median voltage and humidity between the positive and negative voltage groups were tested using the Mann-Whitney *U* test. The Levene’s test was used to compare the variance between the two groups.

## Supplementary Material

Supplemental information can be found online at https://doi.org/10.1016/j.cub.2023.06.021.

Supplementary Materials

## Highlights

- Static charges of animals result in strong electric fields when close to vegetation
- Ticks are pulled by these electric fields across air gaps of several body lengths
- This likely increases the host-seeking efficiency of ticks and other ectoparasites
- Electrostatic attraction of ticks is independent of the host’s charge polarity

## In brief

England et al. show that the naturally accumulated static charges of terrestrial animals are sufficient to passively attract ticks across air gaps of several body lengths of the tick. This electrostatic attraction likely improves the hostseeking efficiency of ticks and many other parasites by increasing the range at which they can attach to hosts.
