# Supplementary material for: Static electricity passively attracts ticks onto hosts
Source: Curr Biol. Author manuscript; Available in PMC 2024 Sep 10. (PMC7616434; doi:10.1016/j.cub.2023.06.021)
Supplement: Supplementary Materials [file EMS198468-supplement-Supplementary_Materials.zip › 1-s2.0-S0960982223007728-mmc1.pdf]

Current Biology, Volume *33*

Supplemental Information

Static electricity passively attracts ticks onto hosts

Sam J. England, Katie Lihou, and Daniel Robert

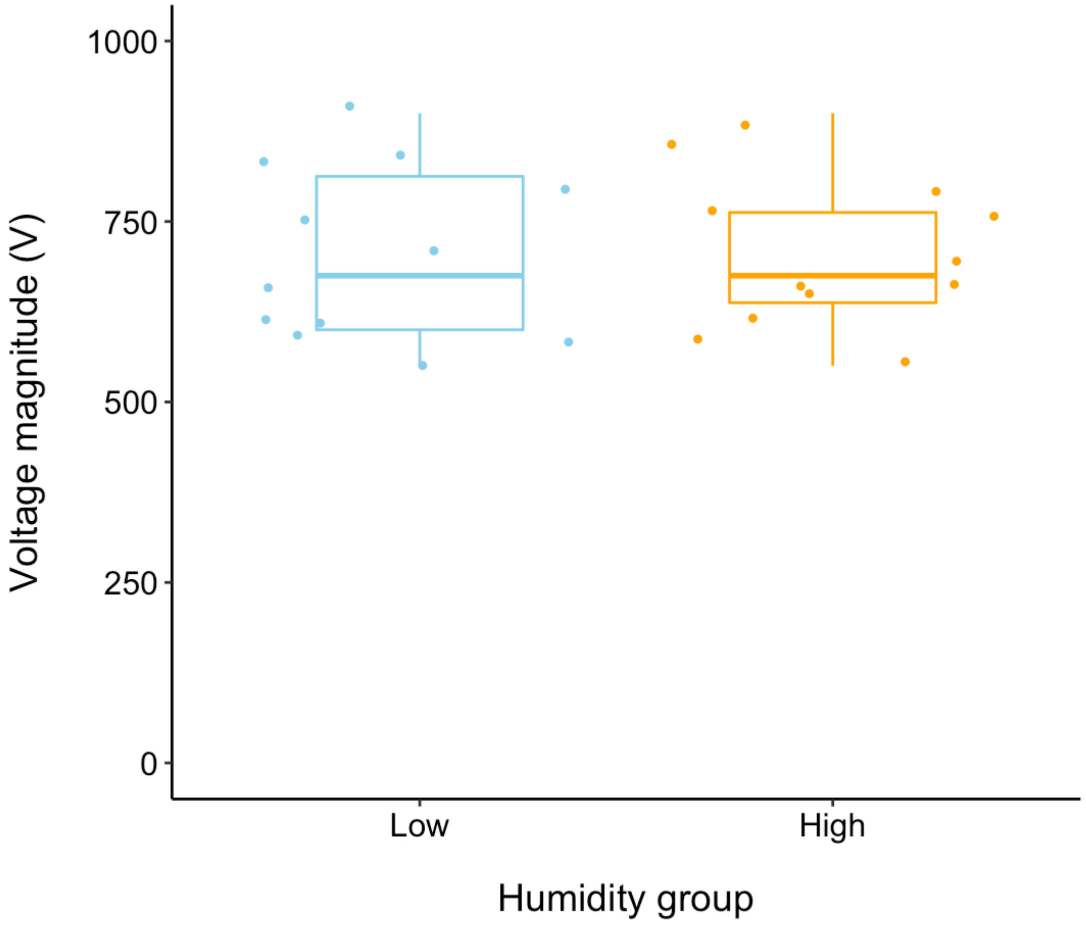

Figure S1: Voltage magnitudes (V) required to fully lift dead ticks across a 2.5 mm air gap against gravity in different humidity conditions. Related to the STAR★Methods. ‘Low humidity’ conditions ranged from 35–39 % (median = 38.5 % (C.I. 38.0-40.5)) relative humidity, and ‘high humidity’ conditions ranged from 59–62 % (median = 61.0 % (60.5–62.0)) relative humidity. Midline of boxplots is the median, edges of boxes are the first and third quartiles, and whiskers are the minimum and maximum, defined by the first and third quartiles ± 1.5 × interquartile range.

Material	Electrical conductivity (S/m)	Relative permittivity	Source(s)
Air	1×10^{-14}	1	[S1]
Soil	0.05	15	[S2,S3]
Plant tissue	0.1	35	[S4–S7]
Animal tissue	0.825	80	[S8]
Wood	2.857×10^{-18}	2	COMSOL
Aluminium	3.774×10^{7}	1	COMSOL

Table S1: List of electrical properties for materials used in the applicable finite element analysis models of electric field strength with sources of numerical values for properties. Related to Figure 2, Figure 3A, and the STAR★Methods.

Supplemental References

S1. Higazi, K.A., and Chalmers, J.A. (1966). Measurements of atmospheric electrical conductivity near the ground. J. Atmos. Terr. Phys. *28*, 327–330. 10.1016/0021-9169(66)90042-0.

S2. Brovelli, A., and Cassiani, G. (2011). Combined estimation of effective electrical conductivity and permittivity for soil monitoring. Water Resour. Res. *47*. 10.1029/2011WR010487.

S3. Rhebergen, J.B., Lensen, H.A., Schwering, P.B.W., Rodriguez Marin, G., and Hendrickx, J.M.H. (2002). Soil moisture distribution around land mines and the effect on relative permittivity. In Detection and Remediation Technologies for Mines and Minelike Targets VII (SPIE), pp. 269–280. 10.1117/12.479098.

S4. Jeon, E., Baek, S., Choi, S., Park, K.S., and Lee, J. (2018). Real-time monitoring of electroconductivity in plants with microscale needle probes. Environ. Control Biol. *56*, 131–135. 10.2525/ecb.56.131.

S5. Ulaby, F.T., and Jedlicka, R.P. (1984). Microwave dielectric properties of plant materials. IEEE Trans. Geosci. Remote Sens. *GE-22*, 406–415. 10.1109/TGRS.1984.350644.

S6. Dadshani, S., Kurakin, A., Amanov, S., Hein, B., Rongen, H., Cranstone, S., Blievernicht, U., Menzel, E., Léon, J., Klein, N., et al. (2015). Non-invasive assessment of leaf water status using a dual-mode microwave resonator. Plant Methods *11*, 1–10. 10.1186/s13007-015-0054-x.

S7. Nadler, A., Raveh, E., Yermiyahu, U., Lado, M., Nasser, A., Barak, M., and Green, S. (2008). Detecting water stress in trees using stem electrical conductivity measurements. Soil Sci. Soc. Am. J. *72*, 1014–1024. 10.2136/sssaj2007.0308.

S8. Zell, M., Lyng, J.G., Cronin, D.A., and Morgan, D.J. (2009). Ohmic heating of meats: Electrical conductivities of whole meats and processed meat ingredients. Meat Sci. *83*, 563–570. 10.1016/J.MEATSCI.2009.07.005.
